# Pneumomediastinum Secondary to Barotrauma after Recreational Nitrous Oxide Inhalation

**DOI:** 10.1155/2016/4318015

**Published:** 2016-11-13

**Authors:** H. Jeddy, Farhan Rashid, H. Bhutta, B. Lorenzi, A. Charalabopoulos

**Affiliations:** ^1^Department of Upper Gastrointestinal Surgery, Broomfield Hospital, Mid Essex Hospital Services NHS Trust, Chelmsford, UK; ^2^Department of Upper Gastrointestinal and Bariatric Surgery, Luton and Dunstable University Hospital, Luton, UK

## Abstract

We present a case of a seventeen-year-old patient, admitted in the care of the surgical team following inhalation of nitrous oxide at high pressure, leading to extensive pneumomediastinum and surgical emphysema. We discuss the subsequent investigations and management for this patient. In the absence of history of airway injury and respiratory problems including asthma and with no oesophageal perforation on investigations, the diagnostic and management challenges encountered have been discussed which will help in future management of similar cases.

## 1. Introduction

Surgical emphysema is the presence of air in tissue. It is due to the breach of an air containing viscous which is in communication with soft tissue and the generation of positive pressure that pushes the air along the tissue plains. Causes of pneumomediastinum and surgical emphysema include iatrogenic airway injuries (e.g., tracheobronchial perforation during intubation, airway injury during chest surgery, ventilator barotrauma, and poorly managed chest drains), asthma (ruptured bullae), oesophageal perforations (iatrogenic or spontaneous), and blunt or penetrating chest trauma [1–5]. The most severe and life threatening cause of such cases, which initiates a significant systemic inflammatory response syndrome and mediastinitis and can lead to multiorgan failure with significant mortality, is a ruptured oesophagus. A ruptured oesophagus should always be included in the differential diagnosis of mediastinitis and surgical emphysema and presents a complex diagnostic and management problem for surgeons/physicians dealing with such cases.

A case of surgical emphysema following high pressure ventilation with nitrous oxide and anaesthesia has previously been reported [1]; however very little has been published following the use of the gas for recreational purposes [2]. We present our experience of a patient who was admitted following inhalation of nitrous oxide at high pressure leading to extensive pneumomediastinum and surgical emphysema.

## 2. Case Presentation

A seventeen-year-old African American male was taking nitrous oxide under high pressure via balloon inhalation at the time of a music festival and also two days prior to the event. He admitted to inhaling approximately fifty balloons worth of gas in total. He had also taken one MDMA (ecstasy) tablet. He noted swelling of the neck with pain and discomfort and presented to the accident and emergency department of the local hospital. The patient denied dysphonia, shortness of breath, and chest pain. On examination his observations were stable. He was saturating at 98% on 2 litres of oxygen. There was marked bilateral neck swelling, extending to the right chest wall and his entire right arm down to his wrist. There was easily palpable crepitus of the aforementioned areas. The airway was central and patent, chest was clear, and there was equal bilateral air entry as well as normal chest wall expansion. Heart sounds were also normal.

### 2.1. Investigations

**Figure 1:**
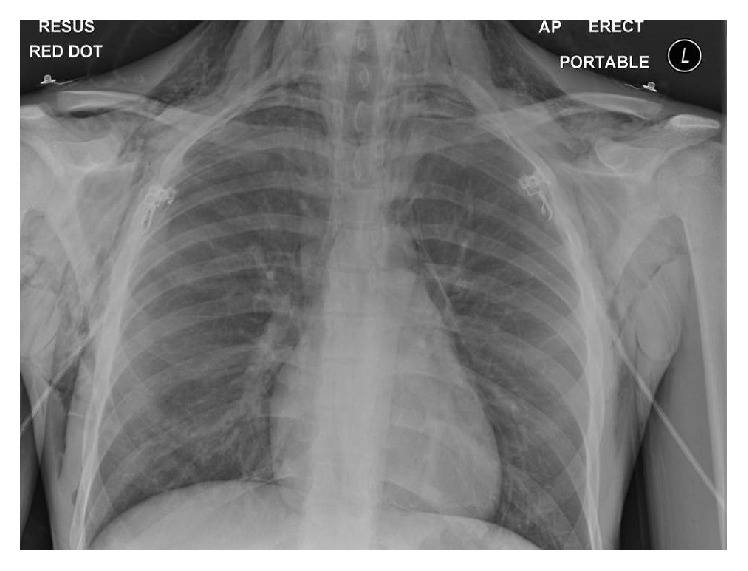
Chest X-ray.

A chest X-ray demonstrated extensive pneumomediastinum of the chest wall and neck, affecting the anterior chest wall, and subcutaneous emphysema can outline the pectoralis major muscle, giving rise to the ginkgo leaf sign (Figure 1).

**Figure 2:**
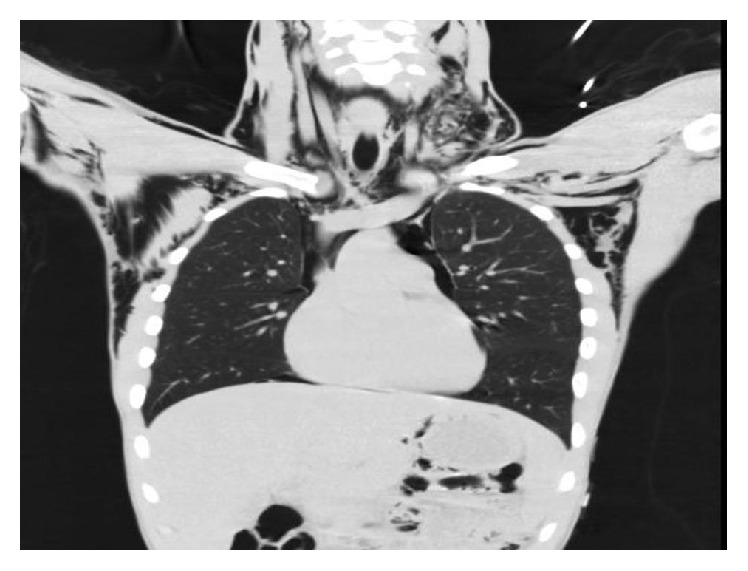
CT scan.

A CT scan (Figure 2) with intravenous contrast confirmed pneumomediastinum and surgical emphysema. The cause of it could not be identified, but there was no obvious tracheal injury or oesophageal rupture apparent. Subcutaneous emphysema is readily visible on CT scans, with pockets of air seen as extremely dark low (air) attenuation areas in the subcutaneous space.

### 2.2. Ongoing Management

The patient was admitted for observation in case of clinical deterioration and potential infection. Although he remained stable and well in himself, the considerable pneumomediastinum seen on initial imaging, combined with concern that this young otherwise healthy patient might be compensating and hence masking a more serious underlying condition, in particular, oesophageal perforation, led to a water soluble (gastrografin) swallow being requested. This revealed no leak of contrast outside the gastrointestinal tract. Given the recent history, a diagnosis of barotrauma with subsequent pneumomediastinum secondary to nitrous oxide inhalation was established.

The patient was discharged home under care of his parents three days after admission. He was strongly advised against future use of the gas for recreational purposes and was told to avoid any contact sports for a duration of six weeks. His subcutaneous emphysema got much better at day 3 on discharge. At 2-week clinic follow-up, it had completely resolved.

## 3. Discussion 

Multiple causes of surgical emphysema and pneumomediastinum have been published [1–4]. Causes of surgical emphysema can be largely divided into three main groups, gas arising internally, gas introduced externally, and gas introduced de novo. Gas arising internally often results from a pneumothorax, pneumomediastinum, or a perforated hollow viscus such as the trachea or oesophagus. Penetrating trauma, postsurgical intervention, and postpercutaneous intervention are examples of when the gas is introduced externally. Gas producing organisms such as those leading to necrotising fasciitis demonstrate production of gas de novo.

Spontaneous pneumomediastinum commonly occurs when an increased intra-alveolar pressure leads to the rupture of the marginal pulmonary alveoli. The air released ascends along the bronchi to the mediastinum and the subcutaneous space of the neck causing cervicofacial subcutaneous emphysema [1]. Surgical emphysema secondary to alveolar rupture from positive pressure ventilation (barotrauma) is a well recognised complication. It is due to excessive manual ventilation and high airway pressure [5].

High airway pressure could have resulted from copious balloon inhalation of nitrous oxide in our patient, leading to rupture of the marginal alveoli.

Nitrous oxide inhalation is a well established anaesthetic technique. It has a long history of safety in medicine and dentistry. However, it is known to cause neurological deficit by reducing the activity of vitamin B12 dependent enzymes in long-term use. The minimum concentration and duration of exposure at which this influence becomes significant are at present not known [5]. Pneumomediastinum has also been reported with MDMA [6]. Since pneumomediastinum has also been reported with MDMA, it is impossible to prove that our patient's pneumomediastinum was due solely to nitric oxide inhalation. With the growing recreational use of the gas as a high in the adolescent population, the threshold of suspicion in young patients who compensate well until the later stages of intoxication and who may have taken other recreational drugs must be high. As this is one of the few cases of surgical emphysema after the recreational use of the gas it is not yet known whether those who have restrictive lung diseases such as COPD and asthma are more prone to alveolar rupture.
